# Exercise addiction in college students: the impact of body dissatisfaction, stress, physical activity and gender

**DOI:** 10.3389/fpsyt.2025.1546192

**Published:** 2025-02-25

**Authors:** Ying Wang, Guohuan Hua, Wenting Liu, Changsheng Wan, Ming Hao, Mingshou Zhang

**Affiliations:** ^1^ School of Public Health and Health Management, Gannan Medical University, Ganzhou, Jiangxi, China; ^2^ School of Resources, Environment and Jewellery, Jiangxi College of Applied Technology, Ganzhou, Jiangxi, China

**Keywords:** exercise risk, sex differences, body dissatisfaction, exercise scores, body fat

## Abstract

**Introduction:**

Engaging in physical activity is commonly regarded as beneficial to health. However, exercise addiction may arise when enthusiasm for exercise reaches a level that disrupts life balance and overall well-being. Factors influencing college students’ exercise addiction remain largely unknown.

**Methods:**

Participants aged 18–23 years (N = 384) underwent body measurements, and sex-adapted silhouettes were employed to assess their level of body dissatisfaction. The Exercise Addiction Inventory was used to investigate the level of exercise addiction among college students. We used t-tests to compare sex differences in BMI, body fat percentage, body dissatisfaction levels, stress levels, and exercise addiction among college students. Chi-square’s test was used to compare differences between males and females in terms of BMI, physical activity levels, stress, and exercise addiction ratings. The exercise addiction level of college students was used as the dependent variable in multiple regression analysis, and BMI, muscle mass, body fat percentage, exercise score, stress level score, and body dissatisfaction level score were used as predictors.

**Results:**

The results of multivariate regression analysis revealed sex differences in physical activity scores, stress levels, body dissatisfaction, and exercise addiction levels, with males consistently exhibiting significantly higher scores than females. In males, body dissatisfaction scores and stress were significant predictors of exercise addiction. Among females, physical activity scores, stress, body dissatisfaction, and body fat percentage were identified as significant predictors of exercise addiction.

**Discussion:**

College students confront risks in relation to exercise addiction. High stress levels and body dissatisfaction may be important causes of exercise addiction. The male students had higher body dissatisfaction, stress levels, and risk of exercise addiction than the female students. With a change in female bodily aesthetics in terms of a shift from a primary focus on thinness-related beauty to having a thin and toned body, the risk of exercise addiction in female college students may increase.

## 1 Introduction

With the rapid progression of social and economic development, many people have gained a deeper awareness of their health, a growing number proactively engaging in sports and seamlessly incorporating physical activity into their daily routines as an integral facet of their lifestyle (1). It is widely acknowledged that consistent and suitable exercise not only improves physical fitness but also mitigates the risk of psychological issues (2). Nevertheless, engaging in inappropriate exercise can be detrimental to health. For certain individuals, exercise can transform into a compulsive pattern, giving rise to excessive activity, which leads to exercise addiction that negatively affects both physical and mental well-being (2).

De Coverley Veale (1987) first introduced the concept of exercise addiction, which refers to the phenomenon wherein an individual’s daily exercise regimen transforms into compulsive behavior, with exercise assuming a paramount role in their lives. Those experiencing exercise addiction may be unwilling to cease exercising even in the presence of injuries, thereby exacerbating their severity (3). Research has demonstrated that individuals dependent on exercise experience adverse emotions such as distress, guilt, and anxiety when they are unable or not permitted to engage in regular exercise routines (4). The subsequent onset of various medical conditions can significantly affect an individual’s quality of life and mental health. Exercise addiction can lead to a spectrum of physiological and psychological disorders including fatigue, sleep disturbances, eating irregularities, and compromised self-control. Notably, withdrawal symptoms similar to those observed in alcohol and drug addictions may manifest in individuals experiencing exercise addiction (3). Not only that, the motivations for exercising differ based on gender (5). The review regarding gender differences in exercise addiction suggests the prevalence of exercise addiction is higher in man compared to women (6). Discerning the roots of exercise addiction and implementing tailored intervention strategies are likely to assist individuals in cultivating a more wholly balanced lifestyle, thereby enhancing their overall quality of life.

Exercise addiction has been suggested to represent a “socially tolerable” manifestation of addiction, propelled by concerns related to appearance anxiety. Additionally, research has indicated that exercise addiction may be rooted in a misperception of body image (7). The results of related meta-analysis finding that the body dissatisfaction is one of the likely causes of exercise addiction (8). Body image can be defined as the mental representation that an individual holds, encompassing perceptions of body size and shape, and associated feelings toward these attributes and bodily components. This construct is intricately linked with historical, social, and cultural factors (9). The media landscape is currently undergoing a rapid evolution, exerting a profound influence on both social culture and individual behavior. People are increasingly becoming more aware of their body image, paying attention to diet, slimming, and forming personal and aesthetic conceptions related to their bodies. The motivation to engage in exercise extends beyond the pursuit of health, and encompasses the desire to regulate body shape, manage weight, and preserve muscle tone through physical activity (10, 11). However, not everyone can have what is perceived as the perfect body under ideal conditions. When there is a discrepancy between ideal and current body shapes, this can trigger dissatisfaction and negative emotions (12, 13). Body dissatisfaction is one of the main risk factors that affect health and motivate individuals to negatively change their life strategies (14). In other words, even with a fit body type, if the current body type is not consistent with an individual’s goal aesthetics, pursuit of that ideal body type may prompt negative emotions (12).

Typically, exercise mitigates or alleviates negative emotions (15). Through exercise, the brain releases neurotransmitters such as dopamine and endorphins, with individuals able to have a physiologically pleasurable emotional experience. A targeted workout can enhance a sense of accomplishment, restore self-confidence, and mitigate negative emotions. However, rapid improvements in body shape through exercise are not always realistic given the influence of genetic factors and other variables (16). Improvements in body shape may not be immediately discernible because of delayed outcomes from exercise. Moreover, with evolving aesthetic perceptions, understandings of the ideal body type may change, contributing to the persistence of body dissatisfaction (17). Such dissatisfaction can further reinforce the pursuit of an ideal body shape, creating a cyclic pattern that can contribute to the development of exercise addiction. Research indicates that individuals exercising for achieving a particular body size are more susceptible to the risk of exercise addiction compared to those engaging in exercise primarily for health promotion (11). Hence, understanding the role of body dissatisfaction may be critical to addressing exercise addiction (18).

College students undergo a demographic transition from adolescence to adulthood (12). This unique period is characterized by numerous physical and psychological changes, highlighting the importance of addressing both physical and mental health challenges faced by college students as they attempt to fulfill their corresponding academic tasks with greater independence. Within this context, the psychological stress on college students is likely to be increased (13). The stress and the related emotions triggered by it are closely associated with exercise addiction, with stress being considered as part of the etiological model of exercise addiction (19). Studies have shown that college students are prone to exercise addiction (6, 20) and that college students have a high level of body dissatisfaction, with gender differences (12, 13). However, few studies have explored the relationships among body dissatisfaction, stress, and exercise addiction among college students. Nevertheless, it is crucial to identify exercise addiction at an early stage and implement interventions for college students displaying signs of excessive exercise, with the aim of enhancing their overall quality of life and mental health.

In summary, body dissatisfaction is a contributing factor to exercise addiction, and college students are a high-risk group for both exercise addiction and body dissatisfaction. However, we know very little about the gender differences in exercise addiction. Therefore, this study aimed to elucidate sex differences in exercise addiction and body dissatisfaction among college students in southern China. Additionally, this study sought to explore the intricate relationship between body dissatisfaction, stress, and exercise addiction in this demographic, understanding the factors influencing exercise addiction among college students and provide a theoretical basis for the implementation of effective targeted intervention measures.

## 2 Methods

### 2.1 Participants

A large comprehensive university located in Jiangxi, China, was selected based on convenience sampling. Jiangxi Province is in southern China. This university is a key educational institution, attracting many students from southern China. Participants were recruited through publicity in students’ dormitory rooms. From September to December 2022, we recruited 400 university students, conducted physical measurements, and completed questionnaires. As there were very few students older than 23 years of age, such students were excluded from the study. Ultimately, 384 students (221 males and 162 females) were fully and effectively included in this study.

### 2.2 Body measurement

Height (0.1 cm precision) was measured using a portable stadiometer (Seca 213, Germany). Body weight (0.1 kg precision), fat percentage, and muscle mass (0.1 kg precision) were measured using a body composition instrument (Tanita BC-610, Japan). These instruments have been widely used in the world. Body mass index (BMI) was calculated based on height and weight (kg/m2), with individuals classified as underweight (BMI < 18.5), normal (18.5 ≤ BMI < 25), and overweight (BMI ≥ 25) (21).

### 2.3 Physical activity

The Physical Activity Rating Scale (PARS-3) was used in this study (22). It assesses the level of physical activity over the past month based on three aspects: intensity, duration, and frequency of participation in physical exercise. Each item is scored from 1 to 5. The total physical activity score is calculated using the formula: intensity × (duration − 1) × frequency, with scores ranging from 0 to 100. Higher scores indicate higher levels of physical activity. The specific classification of physical activity levels is as follows: ≤ol points indicate low physical activity, 20-42 points indicate moderate physical activity, and ≥nd points indicate high physical activity. This scale is widely used globally and by Chinese university students.

### 2.4 Body dissatisfaction

**Figure 1:**
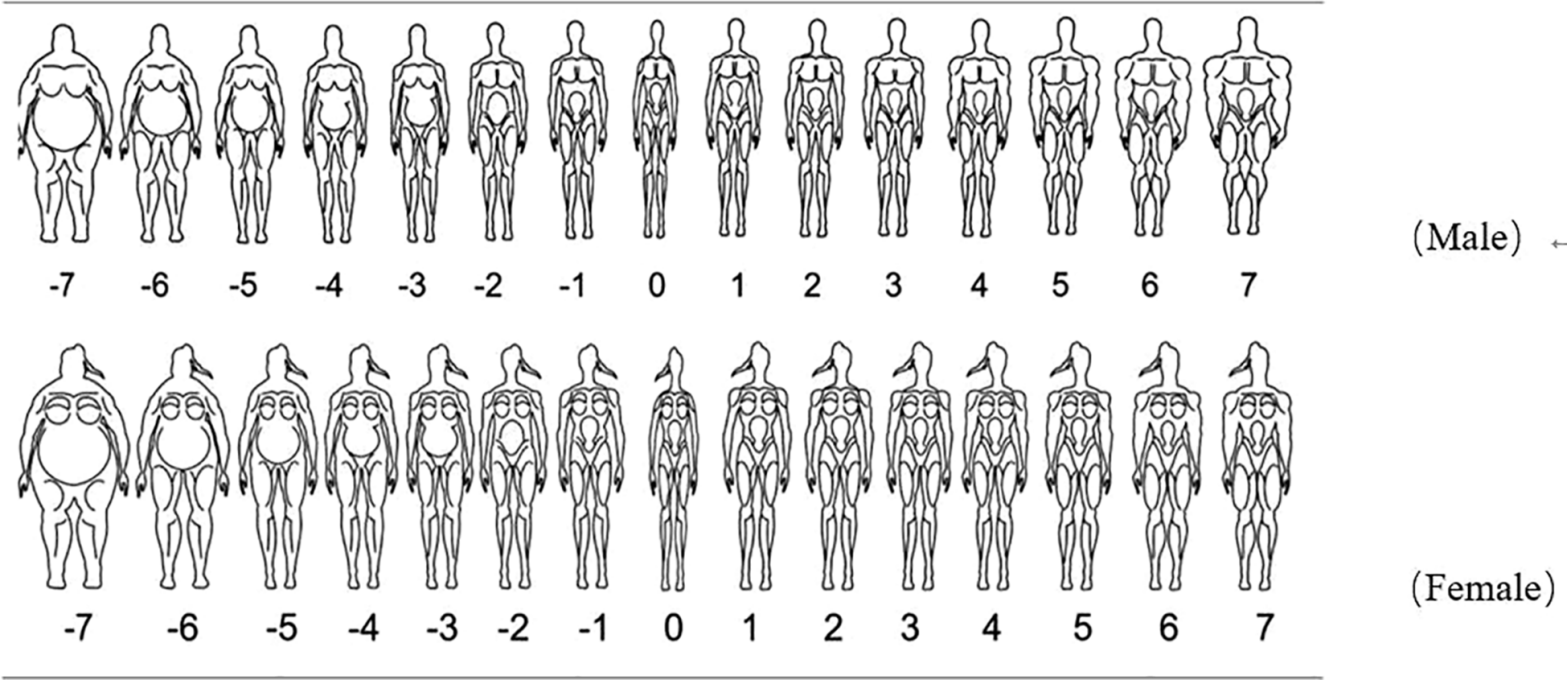
Sex-differentiated silhouettes.

Sex-adapted silhouettes were used to evaluate body dissatisfaction among the college students (**Figure 1**). This scale has proven to have cross-cultural validity and internal consistency and test-retest reliability (21). The images of the set of silhouettes were numbered between -7 (fat) and 7 (muscle). Participants were asked to select the ideal silhouette that they would most like to possess, and the male and female body figures they considered the most attractive. In this study, participants were asked to select their current silhouettes (CS) and ideal silhouettes (IS). The difference between the ideal and current silhouette scores was used to assess the level of body dissatisfaction, allowing the definition of three categorized levels based on quartiles analyses: low dissatisfaction = first quartile (below or equal value 1); median dissatisfaction = second and third quartiles (between values 2 and 4); and high dissatisfaction = fourth quartile (equal or above value 5).

### 2.5 Exercise addiction

Exercise addiction levels of the college students were investigated using the Exercise Addiction Inventory (23), which consists of six items rated on a 5-point Likert scale. This scale has shown good psychometric characteristics across cultures. Individuals with a score of ≥ 24 on the Exercise Addiction Inventory scale were considered to be at risk for exercise addiction, those with a score of 13-23 were considered to have symptoms of exercise addiction, and those with a score of ≤ 12 were considered to have no symptoms of exercise addiction. This scale has been widely used among Chinese university students.

### 2.6 Stress

Stress among college students was investigated using the Perceived Stress Scale (24). The scale includes 14 items to assess the perceived level of stress in various situations in one’s life. The calculation method is to sum the scores of the 14 items, with items 4, 5, 6, 7, 9, 10, and 13 being reverse scored. This scale defines a score of 14 to 28 as low stress, a score of 29 to 42 as medium stress, a score of 43 to 56 as high stress, and a score of 57 to 70 as high stress. This scale has been widely used worldwide.

### 2.7 Statistical analysis

All items were tested for normality and skewness and the data were within acceptable ranges. We used t-tests to compare sex differences in BMI, body fat percentage, body dissatisfaction levels, stress levels, and exercise addiction among college students. Chi-square test was used to compare differences between males and females in terms of BMI category, physical activity levels, stress score category, and Exercise addiction category. The exercise addiction level of college students was used as the dependent variable in multiple regression analysis, and BMI, muscle mass, body fat percentage, physical activity, exercise score, stress level score, and body dissatisfaction score were used as predictors. The gradual increase and decrease method was selected, and the threshold p-value was set to 0.20. All statistical analyses were performed using JMP version 16.0 J software (SAS Institute Inc., Cary, NC, USA). Statistical significance was set at P < 0.05.

## 3 Results

### 3.1 Gender differences and BMI category in body dissatisfaction

**Figure 2:**
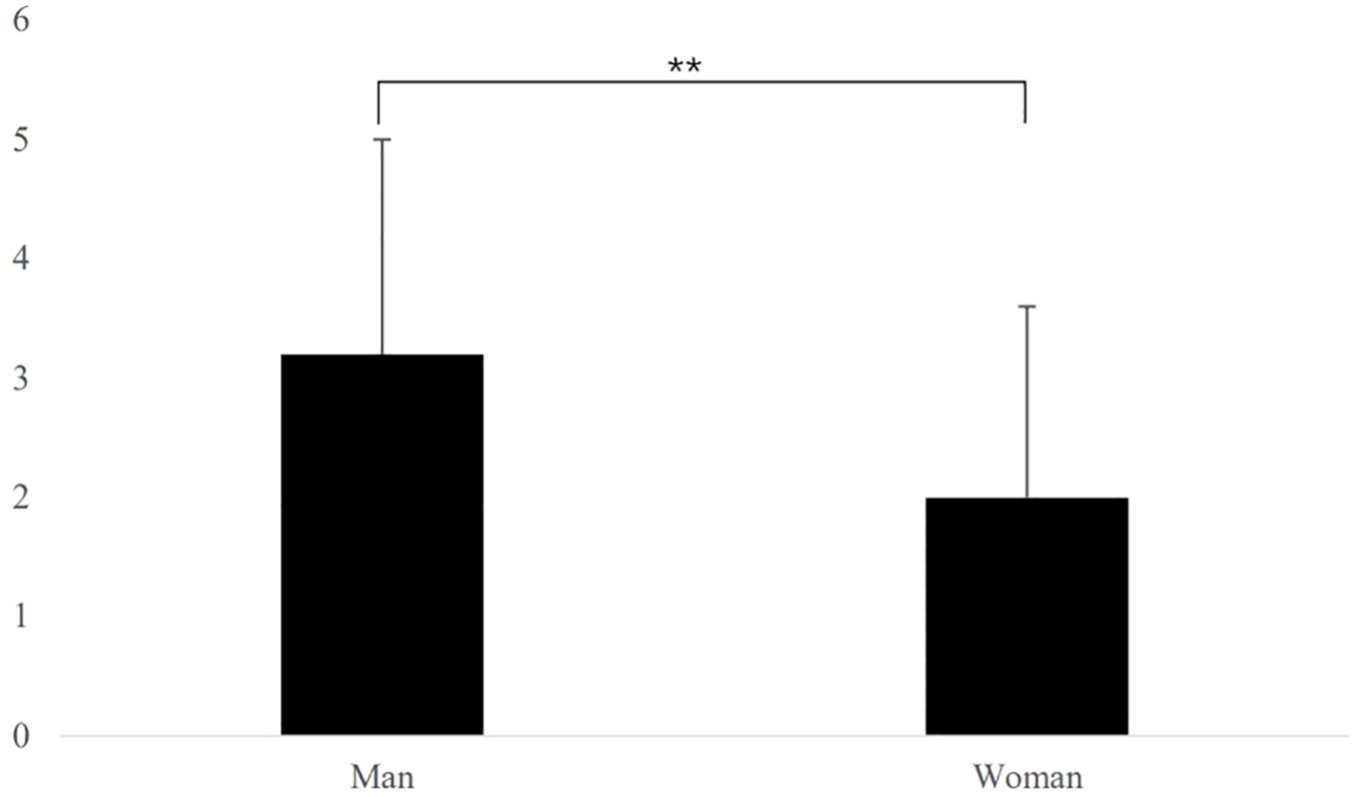
Body dissatisfaction of university students with different sex. **P<0.01.

As shown in **Table 1**, males exhibited a higher BMI and muscle mass than females, whereas females demonstrated a significantly higher body fat percentage than males (P < 0.05). There were sex-related differences in exercise scores, stress levels, body dissatisfaction, and exercise addiction levels. The body dissatisfaction score of males was significantly higher than that of females (P < 0.01), level of body dissatisfaction in males is more distributed between low and high dissatisfaction, while females tend to exhibit more medium levels of body dissatisfaction. (**Table 1**, **Figure 2**).

**Table 1:** Sample characteristics (n=384).

	Mean ± SD or n (%)		P
	Male (221)	Female (162)	
BMI (kg/m2)	22.6 ± 3.5	20.8 ± 2.4	<0.01
Fat%	15.2 ± 6.7	26.8 ± 5.6	<0.01
Muscle mass (g)	52.6 ± 7.5	35.8 ± 3.6	<0.01
BMI category			
Underweight	21 (10)	27 (17)	<0.01
Normal	137 (62)	116 (71)	
Overweight & Obesity	64 (28)	20 (12)	
Physical activity score	18.1 ± 19.5	15.7 ± 17.8	<0.01
Physical activity category			
Low exercise	32 (14)	18 (11)	
Medium exercise	153 (69)	122 (75)	
High exercise	37 (17)	23 (14)	
Body dissatisfaction	3.2 ± 3.3	2.0 ± 2.4	<0.01
Body dissatisfaction category			
Low	130 (59)	82 (50)	
Medium	36 (16)	58 (36)	
High	56 (25)	23 (14)	
Exercise addiction score	16.9 ± 3.8	15.3 ± 3.7	<0.01
Exercise addiction category			
Low	16 (7)	30 (18)	<0.01
Medium	191 (86)	126 (77)	
High	15 (7)	7 (4)	
Stress score	44.0 ± 7.8	37.1 ± 10.9	<0.01
Stress score category			
Low	10 (5)	49 (30)	<0.01
Medium	81 (36)	54 (33)	
Elevated	123 (55)	56 (34)	
High	8 (4)	4 (3)	

BMI, body mass index. The significance of differences between male and female students was determined by t-test (for quantitative variables) or by Chi-square test (qualitative variables).

**Figure 3:**
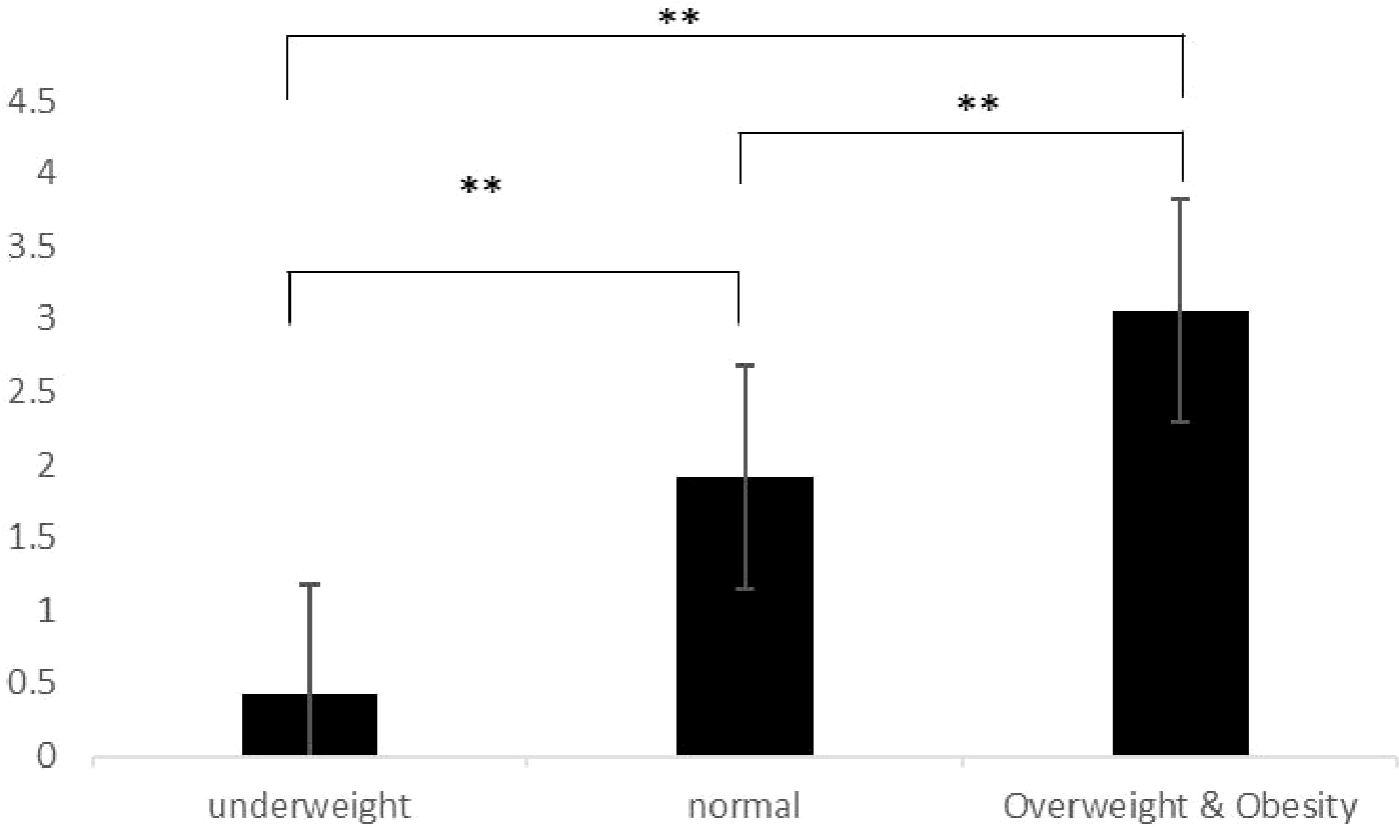
Body dissatisfaction of university students with different BMI category. **P<0.01.

This study examined the differences in body dissatisfaction among different BMI groups using one-way analysis of variance (ANOVA). The ANOVA results showed significant between-group differences (F = 13.182, p < 0.001), indicating that there were significant differences in body dissatisfaction among at least two groups. Further *post-hoc* analysis (LSD method) revealed that the differences in body dissatisfaction between the underweight group and the normal group (mean difference = -1.496, p < 0.01), the underweight group and the overweight & obesity group (mean difference = -2.643, p < 0.001), and the normal group and the overweight & obesity group (mean difference = -1.146, p < 0.01) were all statistically significant. There is a significant association between BMI category and body dissatisfaction, with the overweight & obesity and normal groups showing significantly higher levels of body dissatisfaction compared to the underweight group (**Figure 3**).

### 3.2 Factors influencing exercise addiction

The results of the multiple regression analysis concerning the factors leading to exercise addiction are shown in **Table 2**. For male students, body dissatisfaction (β = 0.43, P < 0.01), exercise score (β = 0.38, P < 0.01), and stress (β = 0.25, P < 0.01) were significant predictors of exercise addiction. For female students, exercise score (β = 0.45, P < 0.01), stress (β = 0.25, P < 0.01), body dissatisfaction (β = 0.15, P < 0.05) and body fat percentage (β = 0.13, P < 0.05) were significant predictors of exercise addiction.

**Table 2:** Factors that contributed to exercise addiction of university students.

	β	t	VIF	P
Male ‡				
IS-CS	0.43	8.96	1.19	<0.01
Physical activity score	0.38	7.91	1.20	<0.01
Stress	0.25	5.63	1.03	<0.01
Female#				
Physical activity score	0.45	7.11	1.10	<0.01
Stress	0.35	5.72	1.04	<0.01
IS-CS	0.15	2.24	1.04	<0.05
Fat%	0.13	2.03	1.26	<0.05

‡ R2: 0.58; P < 0.01; RMES: 2.48.

# R2: 0.43; P < 0.01; RMES: 2.85.

## 4 Discussion

### 4.1 Current status

According to a previous study conducted in the United Kingdom, the level of exercise addiction among non-sports majors was found to be much lower than that among sports majors, with 6.9% of sports majors having exercise addiction compared with only 3.6% of non-sports majors (25). However, it is worth noting that such differences may be narrowing. Findings from a meta-analysis synthesizing data from 34 papers spanning 12 countries and territories indicated that 7% of individuals engaging in exercise generally and 9% of athletes may be susceptible to exercise addiction (26). Notably, exercise addiction has been found to be widespread among Chinese college students. One recent survey of 800 non-sports-major Chinese college students showed that their level of exercise addiction was 5.9% (20). The results of our study showed that the exercise addiction rate of college students majoring in non-sports in southern China was 5.7%, which is similar to the results of that previous study result 5.9%. Therefore, the exercise addiction level of college students majoring in non-sports is also worthy of attention.

### 4.2 The difference between professional athletes and college students

The results of this study showed that, among general college students, the level of exercise addiction among male students was higher than that among female students (**Table 1**). These results differ from those of previous studies on professional athletes. The findings from a pilot study indicated that elite athletes participating in endurance events may exhibit comparable exercise frequencies across sexes (6). By contrast, one study found that professional female marathon runners had significantly higher exercise addiction scores than male marathon runners (27). In contrast to professional athletes who dedicate substantial time and effort to high-intensity professional training for competitive purposes, college students engaging in sports typically operate at a recreational level. Results from a meta-analysis of 27 studies showed that, at the recreational level, males exhibited a higher likelihood of exercise addiction than females (6). Furthermore, it has been concluded that, as females can experience difficulties in relation to their menstrual cycles and that different growth and development processes are involved between men and women, female college students are more likely to approach sports negatively than male college students (28). In summary, male students generally appear to be at a higher degree of exercise addiction, whereas the evidence is inconclusive for differing exercise addiction between male and female professional athletes.

### 4.3 Body dissatisfaction as a significant factor of exercise addiction

The results of this study showed that the higher the level of body dissatisfaction, the higher the level of exercise addiction. The level of body dissatisfaction in males was higher than that in females, with males desiring a stronger body (**Figure 2**). A survey of 632 adult men in the United States revealed that the aspiration for increased muscle mass is a pivotal motivation that drives men to engage in sports, potentially leading to the development of exercise addiction (29). The results of the present study accord with those of previous studies (**Table 2**). However, males and females have different reasons for body dissatisfaction (30, 31). Men aspire for muscle strength, whereas women are more likely to desire a slimmer body shape. The influence of specific beauty standards on female body type is significantly propagated through various media channels, including advertising, fashion magazines, and movies. The aesthetic viewpoint that “thin women are beautiful” would appear to be deeply rooted (32). Thinness in females has become a symbol of beauty, being seen as fashionable, sexy, and as an idealized body form. For many people in modern society, being slim and elegant is considered a condition for females to obtain recognition and benefits (33).

Males who aspire to have greater muscular strength may be more inclined to pursue an idealized body shape through exercise, and body dissatisfaction may be one of the motivations that pushes them to participate more actively in sports. Given males are more likely to want a strong body (31) whereas females are more likely to believe that a slim appearance is beautiful (32), body dissatisfaction among male college students may be an important reason for the higher level of exercise addiction in males than females.

### 4.4 Women and body dissatisfaction

**Figure 4:**
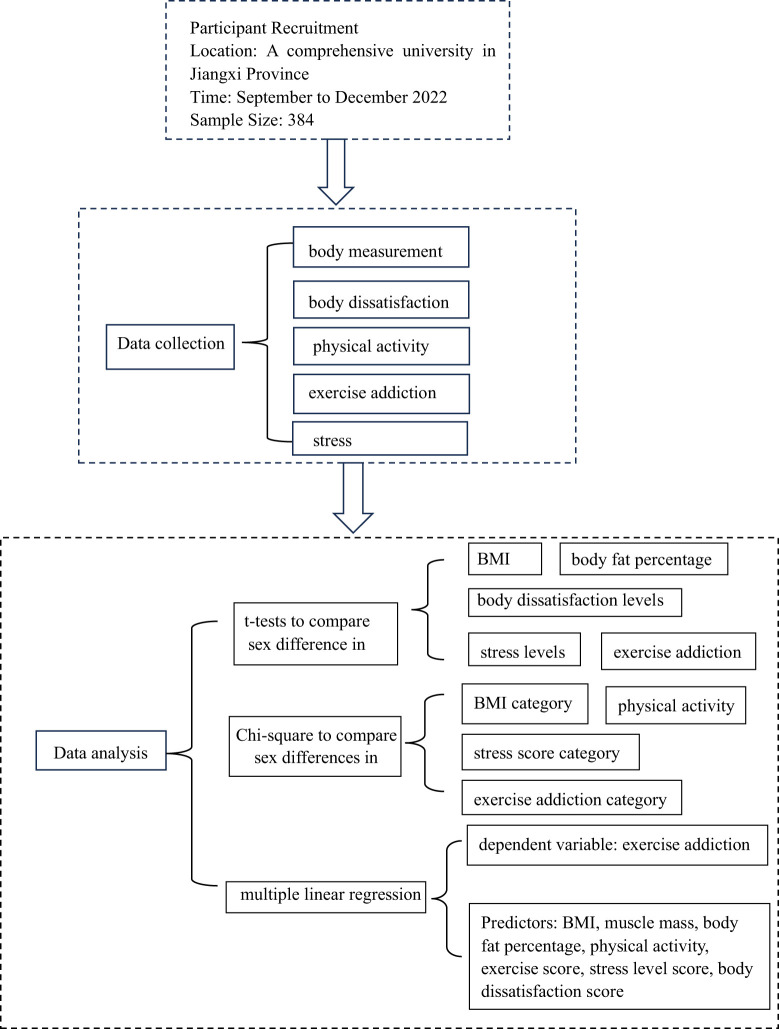
Schematic diagram of the research design.

However, this study also found that some females wanted a toned body (**Figure 4**), which suggests that a certain proportion of females desire to both be slim and develop muscles. Bell in his study of 388 women aged 17 to 35 years in Australia also found similar variations in perceptions of the ideal female body shape (34). Combining slimness and developed muscles is more difficult to achieve than a slim body as greater exercise is required (35). In addition, in Chinese society, perhaps influenced by cultural understandings and expectations concerning marriage, female behavior in terms of body monitoring and body changes is more closely monitored (36). However, a shift in perceptions of an ideal female body shape from being thin to being both slim and fit may increase the likelihood of exercise addiction.

### 4.5 Stress

We also found that exercise addiction among college students might be related to their stress perception. Both male and female college students with higher stress levels had higher levels of exercise addiction (**Table 2**). Numerous studies support the view that exercise is an important way to reduce stress (37, 38). One potential explanation is that exercise reduces the activity in brain areas associated with stress, anxiety, and negative emotions, making exercise a compelling coping strategy for controlling anxiety and worry (38). However, the effect in terms of anxiety reduction may diminish over time, such that the level of exercise needs to continuously increase to achieve the desired effect (37). Additionally, individuals periodically encounter situations in their daily lives that can lead to high stress levels, making it necessary for people with exercise habits to further extend their training programs or increase their exercise intensity to cope (37). It is worth noting that men had higher levels of perceived stress than women (**Figure 4**). Some research suggests that biological differences between men and women may be reflected in mood and behavior and that women may be more sensitive when faced with challenging and uncertain situations (39). Women may be more likely to increase their exercise intensity or duration when faced with daily events that lead to high stress levels, thereby increasing their risk of exercise addiction.

There are some limitations in this study that are worth considering. First, as this was a cross-sectional study, we could not make causal inferences. Second, previous studies have distinguished between primary exercise addiction and exercise addiction secondary to eating disorders. This study did not screen participants with eating disorders. Future research should identify participants with eating disorders and measure their levels of exercise addiction, which may deepen understanding of the pathological processes underlying exercise addiction.

## 5 Conclusion

College students confront risks in relation to exercise addiction. High stress levels and body dissatisfaction may be important causes of exercise addiction. Among the college students in this study, the male students had higher body dissatisfaction, stress levels, and risk of exercise addiction than the female students. With a change in female bodily aesthetics in terms of a shift from a primary focus on thinness-related beauty to having a thin and toned body, the risk of exercise addiction in female college students may increase. This study emphasizes the importance of addressing issues leading to body dissatisfaction and reducing stress in helping to effectively confront the challenge of exercise addiction among college students. Future research should explore the potential mechanisms between stress, body dissatisfaction, and exercise addiction, including whether there are mediating or moderating variables. Longitudinal studies could be designed to track changes in body dissatisfaction and the impact of stress on exercise addiction over time among college students. Additionally, it would be valuable to compare differences in body dissatisfaction, stress, and exercise addiction across college students from different regions or countries.

## Data Availability

The original contributions presented in the study are included in the article/supplementary material. Further inquiries can be directed to the corresponding authors.
